# A Unifying Organ Model of Pancreatic Insulin Secretion

**DOI:** 10.1371/journal.pone.0142344

**Published:** 2015-11-10

**Authors:** Andrea De Gaetano, Claudio Gaz, Pasquale Palumbo, Simona Panunzi

**Affiliations:** 1 CNR-IASI BioMatLab (Italian National Research Council - Institute of Analysis, Systems and Computer Science - Biomathematics Laboratory), UCSC Largo A. Gemelli 8, 00168 Rome, Italy; 2 Sapienza Università di Roma, Department of Computer, Control and Management Engineering (DIAG), Via Ariosto 25, 00185 Rome, Italy; NIDCR/NIH, UNITED STATES

## Abstract

The secretion of insulin by the pancreas has been the object of much attention over the past several decades. Insulin is known to be secreted by pancreatic *β*-cells in response to hyperglycemia: its blood concentrations however exhibit both high-frequency (period approx. 10 minutes) and low-frequency oscillations (period approx. 1.5 hours). Furthermore, characteristic insulin secretory response to challenge maneuvers have been described, such as frequency entrainment upon sinusoidal glycemic stimulation; substantial insulin peaks following minimal glucose administration; progressively strengthened insulin secretion response after repeated administration of the same amount of glucose; insulin and glucose characteristic curves after Intra-Venous administration of glucose boli in healthy and pre-diabetic subjects as well as in Type 2 Diabetes Mellitus. Previous modeling of *β*-cell physiology has been mainly directed to the intracellular chain of events giving rise to single-cell or cell-cluster hormone release oscillations, but the large size, long period and complex morphology of the diverse responses to whole-body glucose stimuli has not yet been coherently explained. Starting with the seminal work of Grodsky it was hypothesized that the population of pancreatic *β*-cells, possibly functionally aggregated in islets of Langerhans, could be viewed as a set of independent, similar, but not identical controllers (*firing units*) with distributed functional parameters. The present work shows how a single model based on a population of independent islet controllers can reproduce very closely a diverse array of actually observed experimental results, with the same set of working parameters. The model’s success in reproducing a diverse array of experiments implies that, in order to understand the macroscopic behaviour of the endocrine pancreas in regulating glycemia, there is no need to hypothesize intrapancreatic pacemakers, influences between different islets of Langerhans, glycolitic-induced oscillations or *β*-cell sensitivity to the rate of change of glycemia.

## Introduction

Over the past 40 years or so, several experimenters have focused their attention on the mechanisms and modalities with which pancreatic *β*-cells secrete insulin in response to glycemic stimuli. Starting with the seminal work of Grodsky [1] experiments have been carried out on explanted animal pancreata, subjected to carefully controlled time-varying glucose concentrations, measuring the resulting insulin secretion [2, 3]. Other experiments have been carried out on animal models (monkeys [4, 5], dogs [6, 7], minipigs [8], rats [9]) as well as human subjects [10–15], administering variable amounts of glucose in different ways, and observing the corresponding insulin serum concentrations.

The results of these many experimental procedures have shown that insulin secretion, in response to glucose stimuli, exhibits a number of diverse and interesting properties, ranging from pulsatility, oscillations, entrainment to exogenous stimuli, first and second phases of release, potentiation, etc. Up to now, when mathematical modelers have been confronted with this diverse array of experimental results, they have concentrated their attention on separate facets of the overall phenomenon. Among the models available in the recent literature, the distributed controller model presented in [16] was however able to reproduce both rapid and slow insulin oscillations, as well as glucose entrainment phenomena: the fundamental idea advanced in that paper was that when a whole population of (heterogeneous) controllers is considered (*firing units*), all of them reacting to the same plasma glucose concentration, then oscillatory phenomena occur, which closely resemble actual experimental observations, and for the emergence of which no oscillatory forcing function (such as oscillating glycolytic glucose degradation or an intrapancreatic pacemaker) need to be assumed. It will be shown in the present work that the same idea can also replicate an extended set of *in vivo* and *in vitro* experiments, provided of course that the model structure is modified in order to reflect the different experiments to be simulated. Indeed, the main result of the present work is to show how a model of this type can account for a wide range of diverse experimental results, using a single set of parameters for the human experiments and a single set of parameters for the rodent experiments. While the model does not include any dependency on the rate of change of glycemia, it will still be shown to reproduce accurately the double phase of insulin release during a prolonged glucose stimulus: a first phase of impulsive insulin release, immediately upon glucose administration, and a second phase of more gradual release, dependent on the *potentiation* effect of the secretory units. Both *in-vivo* and *in-vitro* experimental results will be reproduced: in particular, the *in-vitro* experimental framework under investigation is the one detailed in the pioneering work of Grodsky [1], still considered a standard benchmark to test mathematical models aimed at accounting for the biphasic pattern of insulin release (see, e.g. the works by Bertuzzi, Salinari and Mingrone [17] and by Pedersen et al. [18]).

In addition to its success in replicating such a diverse set of experimental procedures, the proposed approach complies with established physiology, since a population of rather heterogeneous firing units, delivering discrete packets of insulin, is perfectly coherent with the knowledge accumulated on *β*-cells, known to be heterogeneous in their ability to react to glucose [19] and consequently to deliver insulin [20, 21]. It was recently shown [22, 23] that this heterogeneity is preserved over time and that *β*-cells are not identical with regard to the cellular mechanisms that are activated by glucose stimulation, since insulin secretion does not take place simultaneously and at the same rate in all *β*-cells, nor is it a continuous phenomenon, also according to Grodsky [1]. The proposed model is thus conceived so that the independent firing units, while functioning qualitatively in the same way (i.e. obeying equations of the same form), function quantitatively somewhat differently, since their individual model parameters (the glycemic threshold at which they fire, the size of the insulin granules they deliver, their recovery time from the refractory phase, etc.) are not fixed to a single value for all, but are randomly distributed.

Moreover, besides the aforementioned analogies with *β*-cell physiology, the mechanistic behavior of the proposed firing units is similar to that of other cell types in the body (neurons, cardiac and striated muscle cells [24, 25]): all of these cells exhibit some type of critical behavior (impulse transmission, contraction, insulin granule release) when sufficiently stimulated; the critical behavior is then followed by a temporary refractory phase, during which the cell is unable to respond, before response function is progressively restored again (i.e. response occurs at progressively lower stimulation thresholds).

The paper is organized as follows: the next section provides an overview of the most important properties concerning pancreatic insulin secretion, according to different clinical experiments carried out in the last decades, as well as of the state of the art about modeling this secretion. Section 3 describes in detail the distributed population model, stressing the points where its current version differs from the previous version. Section 4 focuses on the experiments the model aims to replicate; a discussion section follows, where numerical results will be provided. Concluding remarks complete the paper.

## Pancreatic insulin secretion: experiments and modeling

Experimental results carried out in the last decades have shown many and diverse features associated with insulin secretion. From a physiological viewpoint, insulin secretion is discontinuous at different scales: at the level of a single *β*-cell, insulin is released as discrete granules, as the final result of the metabolic-electrical activity of the cell, leading to the characteristic behavior known as *bursting*, with a period of tens of seconds from spike to spike (see the work by Pedersen, Bertram and Sherman [26] and references therein); these simple bursts often cluster in compound bursts, with a period of several minutes [27].

Moreover, insulin levels appear to be naturally oscillating *in vivo*, even in the fasting subject. Pørksen et al. [12] argued that pulsatile secretion accounts for 75% of the overall insulin secretion in humans. However, the mechanisms underlying the coordination of the about 1 million of Langerhans islets scattered in the pancreas (as well as of the thousands of *β*-cells collected within each Langerhans islet) to release insulin into short-lived and discrete secretory bursts were not established. There is electrophysiological evidence for coupling of *β*-cells within the same islet [28] and it is known that the oscillatory changes in *β*-cell membrane potential are associated with insulin release [29]: pancreatic neural networks have been hypothesized to modulate and coordinate the pulsatile fashion of the secretion. Indeed, an intra-pancreatic coordinating mechanism, such as an intra-pancreatic neuronal pacemaker, was suggested following experiments that showed preserved pulsatility of insulin release from the isolated perfused pancreas [6, 30–32]. However, it is doubtful that a simple neural network could adequately explain all aspects of such a pacemaker (see the discussion in the work by Matthews et al. [33] and references therein for more detail). Moreover, the comparison of *in vivo* versus *in vitro* experiments shows a substantially different pattern of insulin pulsatile secretion [33, 34] (higher frequency *in vitro*, lower frequency *in vivo*), thus suggesting some further regulation through circulating substrates.

Experiments have shown that glucose concentration oscillates as well, in a fashion strongly correlated with insulin oscillations [4, 10, 35–37]. The question thus arises, whether glucose oscillations are the cause or the consequence of insulin oscillations. Glucose/insulin oscillations tend in fact to exhibit two characteristic regimens, *slow* (period approx. 50–150 minutes) and *fast* (period approx. 5–15 minutes). It was shown that the amplitude and the regularity of the spontaneous slow oscillations in insulin serum concentration were increased when subjects were administered glucose at a steady state (either by constant enteral feeding [13], meal ingestion [38] or constant intravenous infusion [39]). Furthermore, slow oscillations could be entrained by sinusoidally varying intravenous glucose administration rates [14] at frequencies somewhat higher or somewhat lower than the naturally occurring spontaneous frequency. These “ultradian” oscillations have been shown to be substantially independent of the day/night alternation, according to a set of experiments made on night-workers [40]. They also appear to be independent of other ultradian rhythms (e.g. REM-NREM sleep cycle) [41]. Fast insulin serum oscillations could be entrained in their turn by fast pulsing administration of glucose [42], even with amounts of glucose so minute that no variation in glycemia could be detected [15]. A study on fasting conditions in human beings [43] indicates a substantially similar pulsatile pattern according to different fasting periods (10 hours versus 58 hours), with different amounts of overall insulin secretion. In the same paper, another glucose infusion experiment, producing changes in pulse frequency, was considered suggestive of the presence of a glucose-sensitive pacemaker.

Another very interesting feature of insulin secretion is the occurrence of *potentiation*, the ability of the pancreas to respond with progressively increasing insulin amounts to identical glucose stimuli, when these are repeated in close proximity over time [1]. The evident biological value of potentiation is similar to the biological value of immunologic memory: in the one as in the other case the organism reacts more strongly towards a repetition of the (potentially dangerous) stimulus.

A further important feature of insulin secretion, which has attracted considerable mathematical modeling interest, is the *biphasic* reaction to rapidly increasing glycemias: in the first, rapid response phase, *β*-cells secrete what insulin is already docked at the cell membrane in immediately releasable granules, while in the second, delayed phase, new insulin is progressively mobilized from the interior of the cells, packed into granules, which are then docked at the membrane and finally released. This biphasic insulin secretion pattern is particularly evident both in explanted pancreata experiments (starting from the pioneering experiments reported in [1]) and when normal subjects undergo an Intra-Venous Glucose Tolerance Test (IVGTT), with an initial insulin serum concentration spike appearing immediately after the IV administration of the glucose bolus, and a secondary, delayed insulin concentration “hump” depending on sustained pancreatic secretion in the face of glycemias, which do not immediately return to normal. In fact, as the subject’s *insulin sensitivity* declines, e.g. in the progression from normal to prediabetes to Type-2 Diabetes Mellitus (T2DM), the secondary insulin concentration hump is more and more pronounced, given the relative inability of the secreted insulin to force tissues to dispose of the glucose load, leading to sustained hyperglycemia and sustained pancreatic stimulation, combined with a reduced storage of the hormone in docked insulin granules.

Many differential modelling approaches are present in the literature, based on different mathematical structures and aiming to reproduce different features of the glucose-insulin system. In his seminal paper [1], Grodsky offered a summarizing model based on the assumption that insulin is secreted in a discrete fashion, and showed the qualitative similarity of his model predictions with the experimental results he had obtained by stimulating explanted rodent pancreata. Conceptually, the model assumed distributed thresholds, with readily releasable insulin stored in small packets, different packets being associated with different thresholds. Insulin secretion into plasma would occur only when the glucose stimulus exceeded the threshold. This model managed to account separately for both first and second phase insulin release. Grodsky’s distributed threshold model was slightly modified in the work by Overgaard et al. [44], where *in vivo* experiments were considered to validate it. Active and passive insulin compartments were formally defined as the amounts of immediately/not-immediately releasable insulin, respectively, computed by integration of Grodsky’s insulin distribution function for a given glucose level. Another evolution of Grodsky’s work was provided by Pedersen et al. [18], where the insulin distribution function was associated with the Readily Releasable Pool (RRP) of granules. The Authors provide a multicompartmental model, including the RRP, the intermediate pool where granules are primed (and unprimed) to get into the RRP, the fused pool accounting for the insulin secretion rate and a mobilization compartment playing the same role of potentiation as the *provision* in Grodsky’s model. The model accounts also for the so called *kiss-and-run* phenomenon, when fused granules re-seal instead of being released. In another work by Pedersen et al. [45] the Authors show that the model previously developed lends itself to writing the insulin secretion rate as the sum of three terms: one is basal secretion, independent of glucose; the second term is responsible for first phase release, depending on the time-derivative of environmental glucose concentration; the third term is responsible for second phase release, depending on environmental glucose levels. The glycemia time-derivative control term (first phase) is supposed to vanish for smaller and smaller RRP’s (e.g. for diabetic patients). Extensions of the previously cited work by Pedersen et al. [18] have been presented in successive works again by Pedersen et al. [46, 47] for meal ingestion and IVGTT. In a recent work by Pedersen and Cobelli [47], a slight modification in the potentiation mechanism was introduced, with the aim to make the model consistent with available top-down models of insulin secretion. The consequence is that during the IVGTT the glycemia time-derivative term may be neglected. The same philosophy has been followed in [48], where three phases of insulin secretion are considered, similar to those seen in the proportional-integral-derivative type controllers used in engineering control problems.

A different class of models aims to investigate in detail the granule trafficking that determines insulin secretion [17]. These models account for granule formation (from proinsulin and granule material) and for granule diffusion from the reserve pool first into the docked granules compartment, then into the immediately releasable pool of docked granules, and finally into the granules fused with the cell membrane. Exogenous glucose plays a role by modifying the diffusion coefficients from the reserve pool into the docked granules compartment, and those from this last into the immediately releasable pool of docked granules. This model describes a single *β*-cell; nevertheless, overall insulin secretion rate is modeled, in the case of a population of *β*-cells, by defining the fraction of *β*-cells responding to glucose as a saturating function of glucose. The same idea of granule trafficking was expanded by Chen, Wang and Sherman [49] by adding more intermediate compartments from docking to fusion granules and by explicitly taking into account calcium dynamics. This last model was slightly modified by Pedersen and Sherman [50], accounting also for the possibility of exocytosis outside the *L*-type calcium microdomains, for granules with a high sensitivity to calcium. Calcium dynamics is taken, for both models, from [27, 51, 52]. A different molecular model that still exploits the calcium dynamics of the work by Bertram et al. [27], coupled with glycolysis, is the work by Pedersen, Bertram and Sherman [26], where intra- and inter-islet synchronization was investigated with the aim to reproduce the pulsatility of insulin secretion. In the work by Stamper and Wang [53] a recent linear model dealing with 5 compartments of granules is presented.

Other scientists have limited themselves to numerical elaboration of the recorded time-series data, in order to simplify the identification of relevant insulin pulsatility frequencies. Their models offer a high-level mathematical synthetic description of (as yet) unknown or imperfectly understood mechanisms. In this way a formal framework is developed within which it is then easier to formulate specific questions on segments of the overall mechanism; typical in this respect are nonlinear ordinary differential equations models [14] or more recent nonlinear delay-differential equations models [54–56], where a multi-compartment nonlinear system is used to summarize the observable behavior of low frequency insulin oscillations. Models of this type attempt to offer simplified deterministic descriptions of the time course of observed insulinemia, directly relating it to other whole-body state variables (like glycemia), without considering in detail the individual effect of the secretory units (pancreatic *β*-cells, collected in the islets of Langerhans) or the actual molecular mechanisms, which act within the secretory units themselves.

Much modeling has also been done to represent the short time course of glycemia and insulinemia over the few hours following a perturbation experiment: several IVGTT and Oral Glucose Tolerance Test (OGTT) models have been proposed, some with more emphasis on mechanistic, if simplified, interpretation of the shift of substrates [57–60], some including empirical representations, in particular of gastrointestinal absorption of orally administered glucose [61].

None of the aforementioned models is aimed at reproducing the whole framework of clinical experiments reported so far: they were conceived to interpret a specific facet of the overall phenomenon. On the other hand, it is clear that ideally a model of the endocrine pancreas should be able to explain its behaviour by reproducing all available observations simultaneously. Based on the idea of a population of excitable, independent, slightly different controllers, coupled with a simple whole-body model of glucose metabolism and insulin kinetics, the model proposed in the present work will be shown to be sufficient to reproduce well the results of several heterogeneous experimental procedures.

## Materials and Methods

The model presented here is an extension of [16] where, inspired by the seminal work of Grodsky [1], control of glucose-stimulated pancreatic insulin secretion is effected by a discrete set of independent controllers (*secretory, or firing units*), that is, no direct control is exerted on a secretory unit either by other units or by neural or endocrine mechanisms, the only connection among the units being the common input signal represented by blood glucose concentration in *in vivo* situations or environment glucose concentration in *in vitro* experiments, as sensed by each secretory unit. This is consistent with literature data, which stress the crucial role of glucose feedback in governing pulsatile insulin secretion [2, 15, 42]. The physiological identification of the firing unit could be the *β*-cells scattered in the pancreatic Langerhans islet, or, by choosing a different level of model granularity, subcellular granules, or, conversely, collections of synchronized *β*-cells within the islets of Langerhans (the synchronization being essentially due to electrical coupling between neighboring cells) [28, 62]. According to this last interpretation of the firing unit, the total size of the ejected packet may be considered as the total sum of the insulin secreted by all *β*-cells in one islet during a compound burst of excitation [26], i.e. during a fast, isolated series of depolarizations. In any case, we stress the fact that the precise physiological identification of the secretory unit is beyond the scope of the present work.

A previous model [16] had been proposed to account for oscillatory phenomena related to insulin secretion. Here we present a modified version of that model, aiming to encompass a wider set of experiments. Main differences from [16] involve the potentiation effect, which is represented by a second order system (instead of first-order), assuming the loading potentiation rate as a function of the delayed glucose concentration (instead of the current one). Moreover, the glucose threshold distribution of the firing units has been identified by suitably exploiting the experiments in [1] (instead of using a general log-normal distribution).

### Modelling the pancreas

It is assumed that each single secretory unit is able to react to circulating glycemia by ejecting a discrete packet of insulin *J*_*n*_ (pmol/kgBW) if glycemia exceeds that unit’s secretion threshold *B*_*n*_ (mM). When a secretory unit releases its insulin packet, it enters a (relative) refractory state, where further stimulation fails to elicit the release of new hormone. This refractory state is represented in the model by instantaneously increasing that unit’s secretion glycemia threshold to a high level *R*_*n*_ (mM) whence, over time, it exponentially decreases towards its resting threshold value *G*_*n*_ (mM), *G*_*n*_ < *R*_*n*_. The differential equation associated to *B*_*n*_ is:

$$\frac{dB_{n}\left(t\right)}{dt}=-\alpha_{n}B_{n}\left(t\right)+\alpha_{n}G_{n}+\left(R_{n}-B_{n}\left(t\right)\right)\delta \left(\chi (\left\{G\left(t\right)<B_{n}\left(t\right)\right\})\right),$$ (1)

where *α*_*n*_ (min^−1^) is the rate of recovery of sensitivity of the secretory unit, *G*(*t*) (mM) is the external glycemia sensed by all the secretory units, and *δ*(⋅) is a Dirac delta term specifying instantaneous increase of the threshold to the refractory level *R*_*n*_, associated with discharge of insulin, at any time the glucose stimulus *G*(*t*) exceeds the controller threshold *B*_*n*_. In this last expression, *χ* is the characteristic function of its argument set. Notice that simply writing in this equation *δ*(*G*(*t*) − *B*_*n*_(*t*)) would indicate that the controller fires only when glycemia exactly equals its threshold, while the form *δ*(*χ*({*G*(*t*) < *B*_*n*_(*t*)})) indicates that the controller will fire whenever glycemia equals or exceeds its threshold, which is physiologically more plausible. Different values of threshold *G*_*n*_ account for different secretory unit behaviors, with low/high values associated to frequently/seldom firing units. Clearly, the larger is the glycemia *G*(*t*), the larger will be the recruitment of secretory units.

Besides being heterogeneously distributed among the firing units, the releasable insulin packet is assumed to increase in size following prolonged glucose stimulation. Such phenomenon is known as *potentiation* [63, 64], and is represented by the following second-order model:

$$\begin{array}{c} \frac{dJ_{n}\left(t\right)}{dt}=\zeta_{n}\left(D_{n}\left(t\right)-J_{n}\left(t\right)\right)-J_{n}\left(t\right)\delta \left(\chi (\left\{G\left(t\right)<B_{n}\left(t\right)\right\})\right),\\ \frac{dD_{n}\left(t\right)}{dt}=-k_{n}\left(D_{n}\left(t\right)-{\bar{D}}_{n}\right)+\rho_{n}\frac{G^{\gamma_{n}}\left(t-\tau \right)}{G^{\gamma_{n}}\left(t-\tau \right)+\Gamma_{n}^{\gamma_{n}}}, \end{array}$$ (2)

where *J*_*n*_ (pmol/kgBW) is the actual size of the packet for the *n*-th firing unit and *D*_*n*_ (pmol/kgBW) models the current potentiation function level, towards which *J*_*n*_ asymptotically converges at rate *ζ*_*n*_ (min^−1^). The potentiation dynamics (the second equation in Eq (2)) refers to the size of the insulin packet with *k*_*n*_ (min^−1^) being the spontaneous decrease rate of the insulin packet size, *ρ*_*n*_ (pmol/kgBW/min) the maximal loading potentiation rate, Γ_*n*_ (mM) the glycemia at which islet potentiation proceeds at half its maximal rate. The coefficient *γ*_*n*_ determines the progression with which potentiation reacts to circulating glucose concentrations (for small *γ*_*n*_, a moderate potentiation occurs over a wide glycemic range, while for large *γ*_*n*_, abrupt potentiation occurs over a restricted glycemic range). It is supposed that the potentiation function can depend on past values of glycemia, since it is theoretically possible that the glycemic stimulus sensed by the pancreas is delayed, and for this reason a time delay *τ* (min) has been introduced. Finally, the parameter ${\bar{D}}_{n}$ (pmol/kgBW) is the basal insulin packet size towards which the *n*-th firing unit of the *n*-th controller tends at zero glucose stimulus. For instance, assuming glycemia to converge to its basal value, *G*_*b*_, the packet size of a given unit *n* converges to the asymptotic value

$$D_{n}\left(t\right)\to \frac{\rho_{n}}{k_{n}}\cdot \frac{G_{b}^{\gamma_{n}}}{G_{b}^{\gamma_{n}}+\Gamma_{n}^{\gamma_{n}}}+{\bar{D}}_{n}.$$ (3)

As soon as the glucose level in plasma *G*(*t*) exceeds the threshold *B*_*n*_(*t*) of the *n*-th unit, the insulin packet is ejected, and the releasable insulin instantaneously decreases to zero for that unit. The unit then recharges and insulin is stored, in order to be released when needed.

**Remark 1** *The potentiation dynamics is one of the major modifications with respect to* [16]: *the parameter* ${\bar{D}}_{n}$ *had been neglected in the previous model because postulating a zero-glucose stimulus was not reasonable in* in vivo *experiments; on the other hand, when aiming to replicate Grodsky* in vitro *experiments, based on external glucose stimulation of perfused pancreata, it becomes necessary to introduce a non-zero* ${\bar{D}}_{n}$. *The secretory units, in this case, have a basal state (say, at t = t_0_) with each threshold B_n_(t_0_) at the resting value G_n_ and with insulin packet size* $J_{n}\left(t_{0}\right)=D_{n}\left(t_{0}\right)={\bar{D}}_{n}$. *Such a basal value for D_n_ cannot be equal to zero, because experimental evidence shows a clear initial peak of insulin release (the so called first-phase release) at the very beginning of glucose stimulation, even when starting from zero glucose concentration in the perfusate* [1].

Finally, insulin secretion into the portal vein is inherently a discontinuous process, driven by a sequence of Dirac pulses. The Insulin Secretion Rate [ISR (pmol/kgBW/min)] is therefore given by:

$$\text{ISR}\left(t\right)=\sum_{n=1}^{N}J_{n}\left(t\right)\delta \left(\chi (\left\{G\left(t\right)<B_{n}\left(t\right)\right\})\right).$$ (4)

### Modelling the *in vivo* environment

To validate the effects of the proposed insulin secretion model in experiments on living organisms, the same modelling solution of [16] is adopted, and is briefly below reported for convenience. In a living organism, released insulin appears in circulating blood after having gone through the filtering and delaying action of the liver. Such a processes is described by the following *L*-compartmental sub-model:

$$\begin{array}{ccc} \frac{dQ_{1}\left(t\right)}{dt} & = & -h_{d}Q_{1}\left(t\right)-h_{x}Q_{1}\left(t\right)+\sum_{n=1}^{N}J_{n}\left(t\right)\delta \left(G\left(t\right)<B_{n}\left(t\right)\right)\\ \frac{dQ_{2}\left(t\right)}{dt} & = & h_{d}Q_{1}\left(t\right)-h_{d}Q_{2}\left(t\right)-h_{x}Q_{2}\left(t\right)\\ \cdots & & \cdots \\ \frac{dQ_{L}\left(t\right)}{dt} & = & h_{d}Q_{L-1}\left(t\right)-h_{d}Q_{L}\left(t\right)-h_{x}Q_{L}\left(t\right), \end{array}$$ (5)

where *Q*_*i*_ (pmol/kgBW) refers to the insulin amount in the *i*-th compartment, and *h*_*d*_, *h*_*x*_ (min^−1^) are the transfer and clearance rate constants for insulin passage through the liver sinusoids. Simulations (not reported) have shown substantially similar results when coherently varying the number of compartments and the values of parameters *h*_*d*_ and *h*_*x*_. For instance, a larger number *L* of compartments may be compensated by faster compartment dynamics (associated with larger values of *h*_*x*_ and *h*_*d*_). This (*a posteriori*) model unidentifiability is a typical behavior of so-called *s̈loppÿ* models, where a high level of granularity makes it so that many parameter settings are compatible with the output [65]. Therefore, in order to replicate the effect of transit through the liver without getting into the details of organ physiology, here we adopt the solution of a single compartment (*L* = 1). The sloppiness of the model will be further addressed in the Discussion section.

Finally, insulin mass *Q*_*L*_ enters the plasma insulin distribution space according to the following equation:

$$\frac{dI(t)}{dt}=-k_{4}I\left(t\right)+\frac{h_{d}Q_{L}\left(t\right)}{V_{I}},$$ (6)

where *I*(*t*) (pM) is the serum insulin concentration, *k*_4_ (min^−1^) is the linear clearance rate constant for insulin and *V*_*I*_ (ℓ/kgBW) is the apparent distribution volume for insulin.

**Fig 1:**
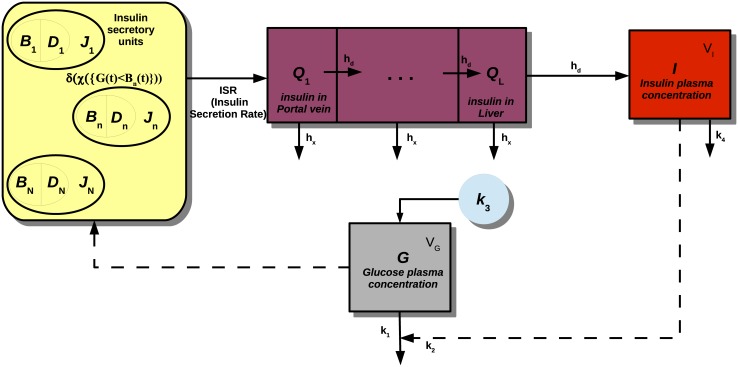
Scheme of the *in vivo* model. The pancreas releases at any given time a total quantity *J*(*t*) of insulin, sum of the amounts *J*_*n*_(*t*) secreted by the single controllers (represented as circles), depending, for the *n*-th controller, on the threshold *B*_*n*_(*t*) and on the potentiation level *D*_*n*_(*t*). Insulin flows from the pancreas through the portal vein (*Q*_1_ compartment) to the liver (*Q*_*i*_ compartments), from which a part of the hormone is lost. Insulin then reaches plasma (*I* is the plasmatic insulin compartment) and stimulates the uptake of glucose by tissues. Glycemia (*G* compartment) is raised by glucose hepatic production *k*_3_ and high glucemias stimulate the production of insulin, closing the cycle.

To describe the dynamic action of insulin on glycemia, a variant of a simple glucose-insulin representation has been used (a block-diagram scheme is reported in Fig 1). Such glucose dynamics was originally introduced by Millsaps and Pohlhausen [66], then incorporated in many subsequent models of the glucose-insulin system [16, 56, 57, 59, 67, 68]:

$$\frac{dG(t)}{dt}=-k_{1}\tilde{u}\left(G\left(t\right)\right)-k_{2}I\left(t\right)G\left(t\right)+\frac{k_{3}\left(t\right)}{V_{G}},$$ (7)

where (i) the first term in Eq (7) describes approximately the (supra-threshold) driving glycemia for urinary glucose elimination:

$$\tilde{u}\left(G\right)=\left\{\begin{array}{cc} 0, & G<G_{u},\\ G-G_{u}, & G\geq G_{u}. \end{array}\right.$$ (8)

with *k*_1_ (min^−1^) the apparent insulin-independent renal elimination rate for glucose, occurring at glycemias greater than the threshold *G*_*u*_ (mM); (ii) the second term is the insulin-dependent glucose uptake, with *k*_2_ (min^−1^pM^−1^) the rate of glucose uptake by tissues per pM of serum insulin concentration; (iii) the third term *k*_3_(*t*) (mmol/kgBW/min) refers to the net balance between hepatic glucose output and insulin-independent zero-order glucose tissue uptake (essentially by the brain), with *V*_*G*_ (ℓ/kgBW) the apparent distribution volume for glucose. In order to account for the noisy time-course of the last term *k*_3_, a stochastic model has been written:

$$k_{3}\left(t\right)={\bar{k}}_{3}+\tilde{s}\left(\xi \left(t\right)\right),$$ (9)

where ${\bar{k}}_{3}$ (mmol/kgBW/min) is a central value for *k*_3_(*t*), *ξ*(*t*) (mmol/kgBW/min) is the stochastic process generated by:

$$d\xi \left(t\right)=-a\xi \left(t\right)dt+bdW_{t},\;\xi \left(0\right)=\xi_{0},\;a,b>0,$$ (10)

with *ξ*_0_ a random variable, independent of the standard Wiener process *W*_*t*_, and $\tilde{s}\left(\cdot \right)$ a suitably defined finite-range function. The parameter *a* (min^−1^) is related to the tendency of the process to return towards zero, while *b* (mmol/kgBW/min^3/2^) determines the volatility of the process. The saturation function $\tilde{s}\left(\cdot \right)$ has been introduced in order to prevent negative or unbounded glucose evolutions, as follows:

$$\tilde{s}\left(x\right)=\left\{\begin{array}{cc} m, & x<m,\\ x, & m\leq x<M,\\ M, & x\geq M, \end{array}\right.$$ (11)

where *m* and *M* (mmol/kgBW/min) are, respectively, the lower and the upper bound of the saturation function.

The model (for human experiments) can be freely accessed from the website http://biomat1.iasi.cnr.it/gemini/pulsatile, where parameter values can be chosen by the user.

### Modelling the *in vitro* environment

The glucose-insulin system exhibits a different behaviour when the pancreas is removed from the living organism and is stimulated to produce insulin by means of externally controlled glucose concentrations. We model the experimental situation described by Grodsky [1], where the pancreas of fasted rats was removed, together with adjacent spleen, stomach and part of the duodenum. The preparation was placed onto a perfusion apparatus and glucose was administered by infusion pump into the celiac artery according to different patterns: the complete effluent was then collected from the portal vein every 30 or 60 seconds and Insulin Secretion Rate (ISR) measured.

In contrast to the *in vivo* environment, in this case the liver is not present and the metabolic loop is not closed by tissue glucose uptake.

**Fig 2:**
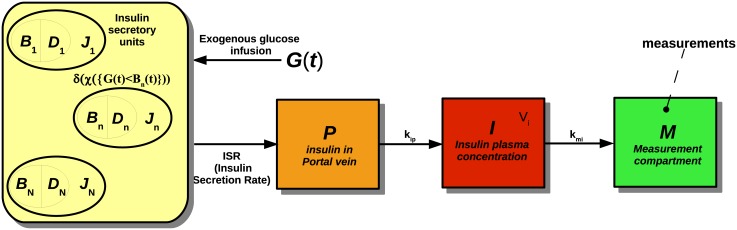
Scheme of the *in vitro* model. Block diagram of the model for the *in vitro* experiments. *B*_1_…*B*_*N*_ and *D*_1_…*D*_*N*_ are respectively thresholds and potentiations of the different controllers, while *J*_1_…*J*_*N*_ indicate the amount of releasable insulin for each controller. *P* represents the portal vein, *I* is the plasma insulin compartment, *M* the measurement compartment. The quantity *G*(*t*) is the external glucose concentration.

The *in vitro* environment is diagrammed in Fig 2: the pancreas releases insulin into the portal vein (*P* compartment), then the hormone flows into the plasma compartment *I* and eventually into the measurement compartment *M*. The equations describing this process are:

$$\begin{array}{c} \frac{dP(t)}{dt}=\sum_{n=1}^{N}J_{n}\left(t\right)\delta \left(\chi (\left\{G\left(t\right)<B_{n}\left(t\right)\right\})\right)-k_{ip}P\left(t\right), \end{array}$$ (12)

$$\begin{array}{c} \frac{dI(t)}{dt}=k_{ip}P\left(t\right)-k_{mi}I\left(t\right), \end{array}$$ (13)

$$\begin{array}{c} \frac{dM(t)}{dt}=k_{mi}I\left(t\right), \end{array}$$ (14)

where *k*_*ip*_ (min^−1^) is the apparent transfer rate constant between portal and serum insulin compartments, and *k*_*mi*_ (min^−1^) is the apparent transfer rate constant between serum and measurement compartments. The reason of the choice of three compartments downstream of the pancreas is due to the need to replicate the observed shape of the sudden increase of ISR as shown in Fig 1 of the work by Grodsky [1], during the external administration of a constant quantity of glucose.

According to [1], the ISR in *in vitro* experiments is obtained by measuring the amount of insulin produced during a time interval, and dividing it by the elapsed time (typically, 30 seconds or one minute):

$$\text{ISR}\left(t\right)=\frac{M(t+\Delta t)-M(t)}{\Delta t},\;\Delta t=0.5\;\text{min}.$$ (15)

In the numerical simulations, the integration time interval has been set to Δ*t* = 0.5 min for the simulation of the *in vitro* experiments, and to Δ*t* = 0.1 min the simulation of the *in vivo* experiments.

## Results

This section shows how a variety of experiments reported in the literature can be faithfully reproduced by the proposed model. These experimental procedures encompass both *in vivo* and *in vitro* experiments, giving rise to both fast and slow oscillations in insulinemia, and showing the typical biphasic response of insulinemia when a bolus amount of glucose is rapidly administered.

It is to be underscored that all the simulations described in the present work have been generated using the same set of parameter and meta-parameter values, possible changes reflecting the species of the experimental subject and the mechanics of the experimental procedure itself. In fact, the simulations assume a generic human subject (whose parameters and meta-parameters are shown in Table 1) or a rat (whose parameters and meta-parameters are shown in Table 2). Even though the model requires the evolution of thousands or even millions of firing units, hence thousand or millions of coupled equations, the set of involved (meta)-parameters is small and the model does not exhibit the *sloppiness* property, such as usually appears in ODE molecular models when many reactions/equations are coupled together, with possibly hundreds of independent parameters. This is a crucial point: even though the current model is composed of thousands or millions of equations, its parameter set is small with respect to the information content of the experiments and the model is therefore identifiable from available data. In other words, in the situation discussed here it is not the case that the high dimensionality of the parameter set easily allows the model to fit any data set. In fact, the contrary is true: the data sets are so diverse, their morphology is so rich, and the parameter set is so relatively small, that if the model structure were not correct a good fit would be unattainable.

**Table 1:** Parameter values for the *in vivo* model.

Symbol	Units	Mean	Std. deviation	Value
*N*	#	–	–	100’000
*L*	#	–	–	1
*τ*	min	–	–	8
*h*_*d*_	min^−1^	–	–	0.9
*h*_*x*_	min^−1^	–	–	0.5
*k*_1_	min^−1^	–	–	8.57 × 10^−3^
*k*_2_	min^−1^pM^−1^	–	–	1.4 × 10^−4^
${\bar{k}}_{3}$	mmol/kgBW/min	–	–	0.01
*k*_4_	min^−1^	–	–	0.08
*V*_*I*_	l/kgBW	–	–	0.25
*V*_*g*_	l/kgBW	–	–	0.2
*G*_*b*_	mM	–	–	4.25
*α*_*n*_	min^−1^	0.3	0.12	–
*R*_*n*_	mM	1000	50	–
*k*_*n*_	min^−1^	0.1	0.002	–
${\bar{D}}_{n}$	pmol/kgBW	0.003	3.6 × 10^−5^	–
*ρ*_*n*_	pmol/kgBW/min	6.5 ×10^−3^	2 × 10^−6^	–
Γ_*n*_	mM	9	0.1	–
*γ*_*n*_	#	10	0.1	–
*ζ*_*n*_	min^−1^	0.1	0.02	–
*ν*	#	–	–	2.5137
*g*_1/2_	mM	–	–	9.7697
*k*_*ga*_	min^−1^	–	–	0.03
*a*	min^−1^	–	–	0.04
*b*	mmol/kgBW/min^3/2^	–	–	5.56 × 10^−4^
*m*	mmol/kgBW/min	–	–	−5.56 × 10^−3^
*M*	mmol/kgBW/min	–	–	9.44 × 10^−2^
*G*_*u*_	mM	–	–	9

**Table 2:** Parameter values for the *in vitro* model.

Symbol	Units	Mean	Std. deviation	Value
*N*	#	–	–	10’000
*τ*	min	–	–	0
*k*_*ip*_	min^−1^	–	–	0.85
*k*_*mi*_	min^−1^	–	–	4.8
*α*_*n*_	min^−1^	0.65	0.4	–
*R*_*n*_	mM	1000	50	–
*k*_*n*_	min^−1^	0.1	0.002	–
${\bar{D}}_{n}$	pmol/kgBW	0.09	5 × 10^−4^	–
*ζ*_*n*_	min^−1^	0.2	0.02	–
*ρ*_*n*_	pmol/kgBW/min	3.2 ×10^−2^	2 × 10^−5^	–
Γ_*n*_	mM	10	0.1	–
*γ*_*n*_	#	5	0.05	–
*ν*	#	–	–	2.5137
*g*_1/2_	mM	–	–	9.7697
*k*_*ip*_	min^−1^	–	–	0.8
*k*_*mi*_	min^−1^	–	–	4.8

The aim to reproduce more clinical experiments than the ones considered in [16] led us to modify some of the equations of the model (such as the ones related to potentiation) and to perform again the whole process of parameter assessment. The new model (endowed with the new set of univocally fixed parameters) had of course to faithfully replicate the new experiments as well as the old ones. This fact could not be taken for granted, since the model was changed: this is the reason why the new simulations performed on the old experiments, that had already been considered in [16], have also been reported.

From a numerical viewpoint, one important question was how many secretory units should be considered for experiments on human subjects. To this end, starting from a minimal set of *N* = 10^3^ secretory units, we looked at the output variations occurring as *N* increased over several orders of magnitude, like 10^4^, 10^5^, 10^6^, etc. The total amount of available insulin, produced by the *N* secretory units, was however kept fixed when increasing *N*, to make comparisons possible. The results of our simulations show that no significant changes occur in the output profiles by increasing *N* beyond 10^5^. This fact allows us to distinguish between the physiological meaning of a secretory unit from its numerical simulation meaning. In other words, by considering as firing units 10^9^ pancreatic *β*-cells or 10^6^ islets of Langerhans (each possibly working as a single firing unit due to *β*-cells synchronization) does not produce appreciably different results in the corresponding numerical simulations.

A further concern involves the statistical variability among different samples of secretory units (of same sample size *N*) from the same population (identified by a single set of meta-parameters). The simulations have shown no substantial modifications in the main features of the glucose-insulin evolutions across different same-sized samples drawn from the same population, thus supporting the consistency of the numerical approach: the glucose-insulin profiles are emergent properties of the chosen meta-parameters, rather than occasional results due to random outliers.

The rremainder of this section is structured as follows: the first part shows the simulation of *in vitro* experiments as performed by Grodsky [1]. The second part reproduces slow and fast insulin oscillations (as performed respectively by Simon et al. [13] and by Pørksen et al. [15]), as well as the entrainment of the insulinemic signal (as performed by Sturis et al. [14]) in man. Finally, simulated intra-venous glucose tolerance tests (IVGTTs) are carried out on virtual normal, pre-diabetic and Type 2 Diabetes Mellitus (T2DM) virtual subjects.

### Biphasic insulin secretory response in a perfused rat pancreas: Grodsky 1972

We report here simulations of the experiments performed by Grodsky et al. [1], with different patterns of glucose concentration stimulating insulin production by explanted, perfused rodent pancreata. Some preliminary results have been presented in [69].

The mathematical model is described by Eqs (1)–(3) and (12)–(14). Given the number *N* of secretory units (in case of pancreata coming from rodent, *N* has been set to 10^4^), one has a system of (3*N* + 3) ordinary differential equations. The distributions chosen for the parameters, except the one related to the resting threshold *G*_*n*_, are independent log-normals (with means and standard deviations reported in Table 2). The distribution of the resting threshold *G*_*n*_ has been reconstructed from the data in Fig 2 of the original work by Grodsky [1], where the insulin secretion rate stimulated by an exogenous stepwise glucose administration in the range from 50 mg/dl up to 500 mg/dl is reported. Since the glucose stimulus is delivered starting from zero resting glycemia, it can be hypothesized that the initial peaks are due to all the recruitable secretory units at the given glycemic level: all units should in fact be at their resting value (*B*_*n*_(0) = *G*_*n*_) before the infusion is administered (*G*(*t* < 0) = 0). Therefore, Table 3 can be constructed, where the Cumulative Distribution Function (depending on the glucose concentration *g*), obtained by ordinary least squares fitting, can be empirically inferred as:

$$F\left(g\right)=\frac{g^{\nu }}{g_{1/2}^{\nu }+g^{\nu }},$$ (16)

with

$$\nu =2.5137,\;g_{1/2}=9.7697\;\text{mM}.$$ (17)

The derivation of the corresponding probability density *f*(*g*) is straightforward:

$$f\left(g\right)=\frac{dF(g)}{dg}=\nu g_{1/2}^{\nu }\frac{g^{\nu -1}}{{\left(g^{\nu }+g_{1/2}^{\nu }\right)}^{2}}.$$ (18)

**Fig 3:**
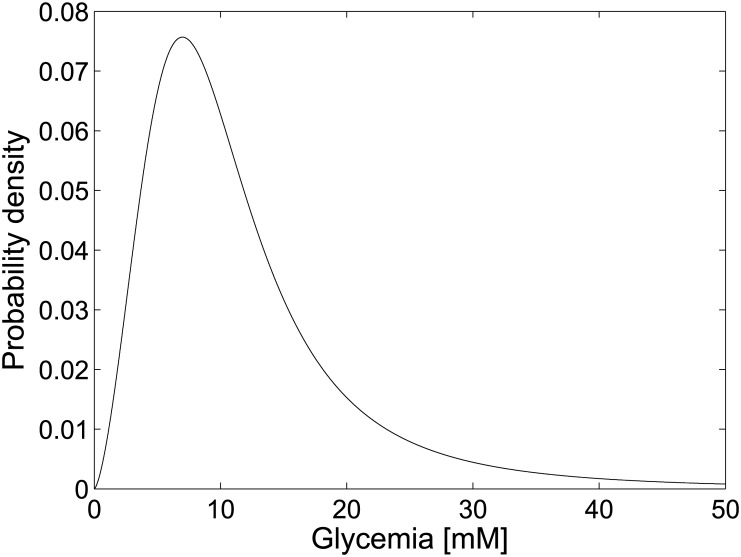
Probability density function of the controller firing thresholds *G*_*n*_.

This density has been plotted in Fig 3: from it *G*_*n*_ samples can be drawn.

**Table 3:** Percent activation (PA) of pancreatic *β*-cells subject to different glycemic stimuli, as extracted from Fig 2 in [1].

Glyc. (mg/dl)	50	100	150	200	300	500
**PA**	2%	18.75%	43.75%	56.25%	75%	100%

The other (meta)-parameters for rodents have been calibrated to best reproduce the following five experiments from Grodsky [1].

**Fig 4:**
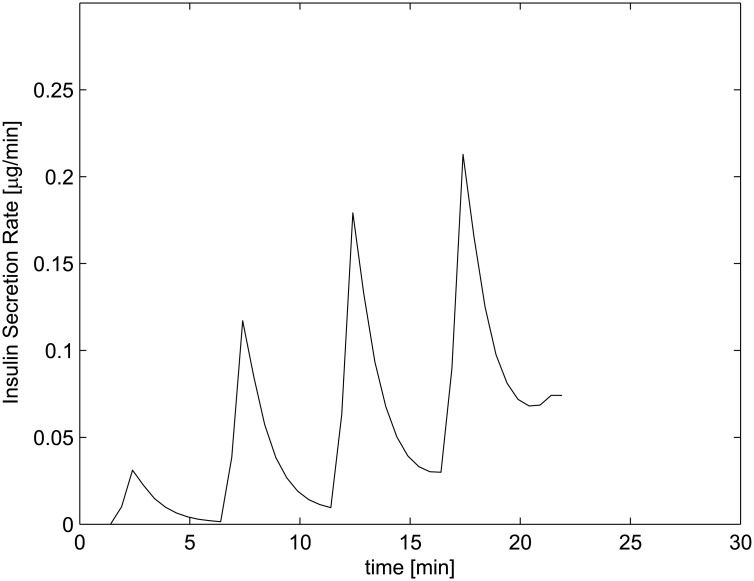
Insulin Secretion Rate at staircase-increasing glucose concentrations, comparable with Grodsky’s first experiment (Fig 1 in [1]).

The first experiment consists of a staircase stimulation with glucose administered at 50 mg/dl increments every 5 minutes, from 50 mg/dl up to 200 mg/dl. The experiment focuses on the first phase of pancreatic action, that is the initial spike of ISR. In fact, not enough time passes between successive glucose administration steps for insulinemia to show a secondary slow increment. The model reproduces remarkably well the observed results, both as regards the timing of the spikes, their size increments and the persisting increased insulin secretion rate at the end of each spike and until the next (due to the irregular activity of larger and larger proportions of the recruitable set of secreting units): compare Fig 4 with Fig 1 in Grodsky [1].

**Fig 5:**
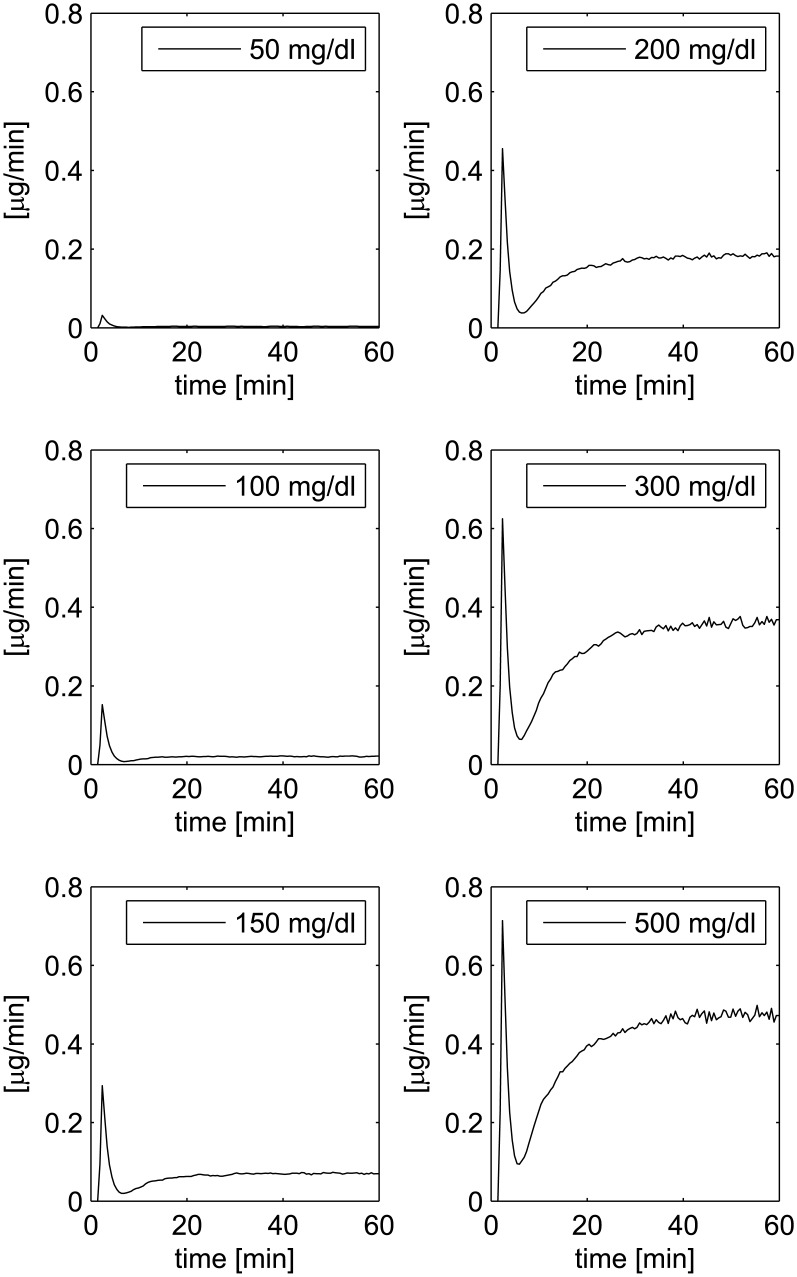
Insulin Secretion Rate at different levels of constant glucose administration, comparable with Grodsky’s second experiment (Fig 2 in [1]).

The second experiment shows both rapid and slow phases of insulin release: different constant glucose infusions (50, 100, 150, 200, 300 and 500 mg/dl) are administered and the insulin secretion rate is measured over 60 minutes. The proposed model replicates accurately this experiment, as shown in Fig 5, comparable with Fig 2 in Grodsky [1].

**Fig 6:**
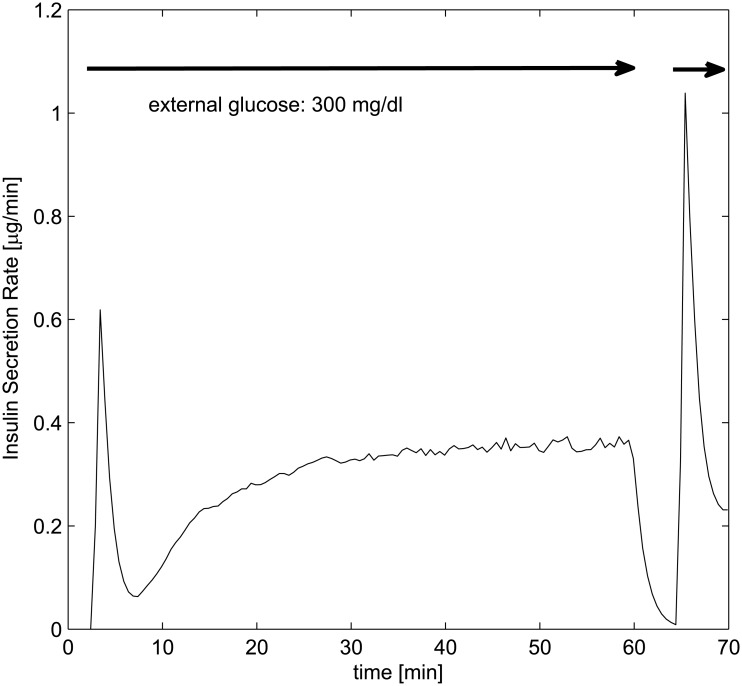
Insulin Secretion Rate for discontinuous constant glucose administration, comparable with Grodsky’s third experiment (Fig 3 in [1]).

The third experiment consists of a sustained, intense glucose stimulation at 300 mg/dl for 60 minutes, followed by removal of any external stimulus for 5 minutes, and final reestablishment of the same level of stimulus. This experiment shows specifically the role played by potentiation: in fact, due to the persisting glucose stimulus, the islet are progressively potentiated (that is, in the model, *D*_*n*_(*t*) increases and consequently the packet size *J*_*n*_(*t*) of the *n*-th secretory unit becomes larger). When the stimulus is removed and then re-administered (before potentiation is given the necessary time to return to baseline), the peak of ISR is much larger in amplitude than the initial one. Fig 6 shows the result of the simulated experiment: comparing it with Fig 3 in Grodsky [1] the similarity of original and simulated tracings is striking.

**Fig 7:**
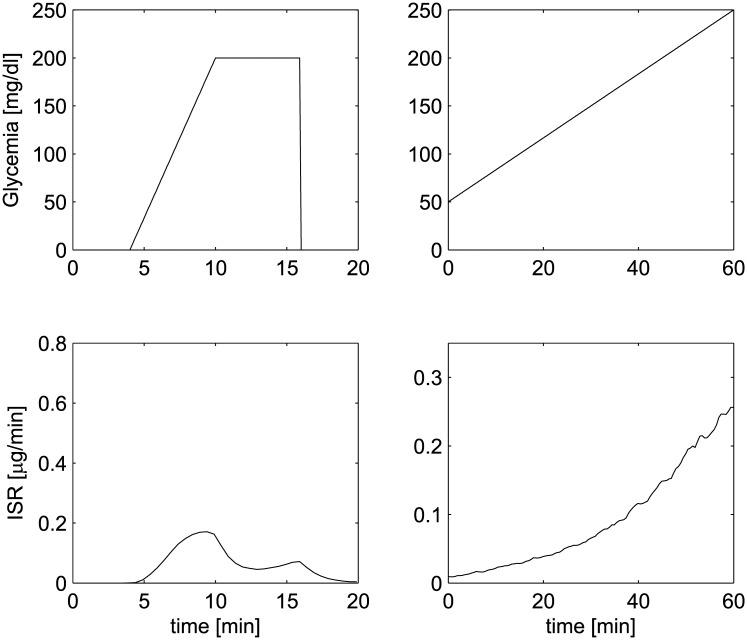
Insulin Secretion Rate when a pattern of increasing glucose concentrations is reproduced, comparable with Grodsky’s fourth and fifth experiments (Figs 4 and 5 in [1]).

The fourth and the fifth experiments show the pancreatic response when external glucose is administered at a constant trending rate; the fourth experiment consists of a combination of ramp followed by maintenance of a constant level of glucose concentration, while in the fifth experiment a continuously increasing stimulus (ramp) is applied. The effect of potentiation is therefore damped in the first case, due to the fact that, though present during the increasing phase of the stimulus, it is not further strengthened during the constant administration, when, conversely, the previously excited secretory units are not able to recover from their refractory state. This determines the observed drop in ISR. In the last experiment, on the other hand, since the stimulus is always increasing, the potentiation effect combined with the progressively larger recruitment of secretory units causes a constant increase in ISR. The results of the simulated experiments are reported in Fig 7, comparable with Figs 4 and 5 in Grodsky [1].

### Ultradian oscillations in man: Simon et al. 1987

We will henceforth use a different set of model (meta)-parameters (Table 1) in order to represent the glucose-insulin system in man. Lacking information on the distribution of firing glycemia thresholds in humans, we adopted, at least provisionally, the same distribution identified in rats, as represented in Fig 3; regarding the position of the distribution, we suppose it to be approximately the same in rodents and humans, since it is reasonable that a healthy pancreas manages normal glycemic levels with a fraction of the available islets (i.e. those which have $G_{n}<\bar{g}$, with $\bar{g}$ the daily mean glycemia), leaving the others free to intervene in case of need, with a large hormone secretion reserve to be used, for instance, after meals.

In this paragraph, the experiment performed by Simon et al. [13] is described and the results of its *in silico* reproduction with our model is shown. The experiment shows how slow (*ultradian*) spontaneous oscillations in insulinemia become more evident when a patient undergoes continuous, constant enteral feeding.

In [13] the exogenous glucose perturbation was a continuous enteral nutrition of 90 kcal/h, composed of 50% carbohydrate, 35% fat and 15% protein. Glycemia and insulinemia samples were collected over a 24-h time-period, with a sampling time of 10 min. Since enteral nutrition consists in delivering the meal (a mixture of proteins, carbohydrates, fats, vitamins and minerals) through a tube directly into the stomach or small bowel, a simple first-order gastro-intestinal tract model has been adopted:

$$\frac{dA(t)}{dt}=-k_{ga}A\left(t\right)+v_{\text{ent}}\left(t\right),$$ (19)

where *A*(*t*) (mmol/kgBW) is the glucose mass in the splanchnic compartment, *k*_*ga*_ (min^−1^) is the splanchnic glucose absorption rate, and *v*_ent_ (mmol/kgBW/min) is the enteral glucose infusion rate. This input term has to replicate the glucose effectively provided by the administered enteral nutrition, and for this reason we assume that carbohydrates, proteins and fats either directly provide glucose, or indirectly (e.g. via Randle’s cycle) spare glucose entry into the Krebs cycle. Considering Eq (19), plasma glycemia Eq (7) thus becomes:

$$\frac{dG(t)}{dt}=-k_{1}u\left(G\left(t\right)\right)+k_{2}I\left(t\right)G\left(t\right)+\frac{k_{3}\left(t\right)+k_{ga}A\left(t\right)}{V_{G}}.$$ (20)

Regarding the value of the input *v*_ent_, a rough simplification consists therefore in considering all administered calories as representing (possibly delayed) glucose administration:

$$90\;\text{kcal/h}≃22.5\;\text{g/h}≃\frac{22,500}{60}\;\text{mg/min}≃375\;\text{mg/min}.$$ (21)

Taking into account an average (male, female) normal body weight of 60 kg, we compute that a reasonably approximate “glucose” enteral administration rate could be:

$$v_{\text{ent}}\left(t\right)≃6.25\;\text{mg/kgBW/min}≃0.035\;\text{mmol/kgBW/min}.$$ (22)

Following Simon et al. [13], intra-assay coefficients of variation have been assumed for glycemia (= 1%) and insulinemia (5.8%), and both signals have been finally filtered by means of a three-point moving average low-pass filter.

**Fig 8:**
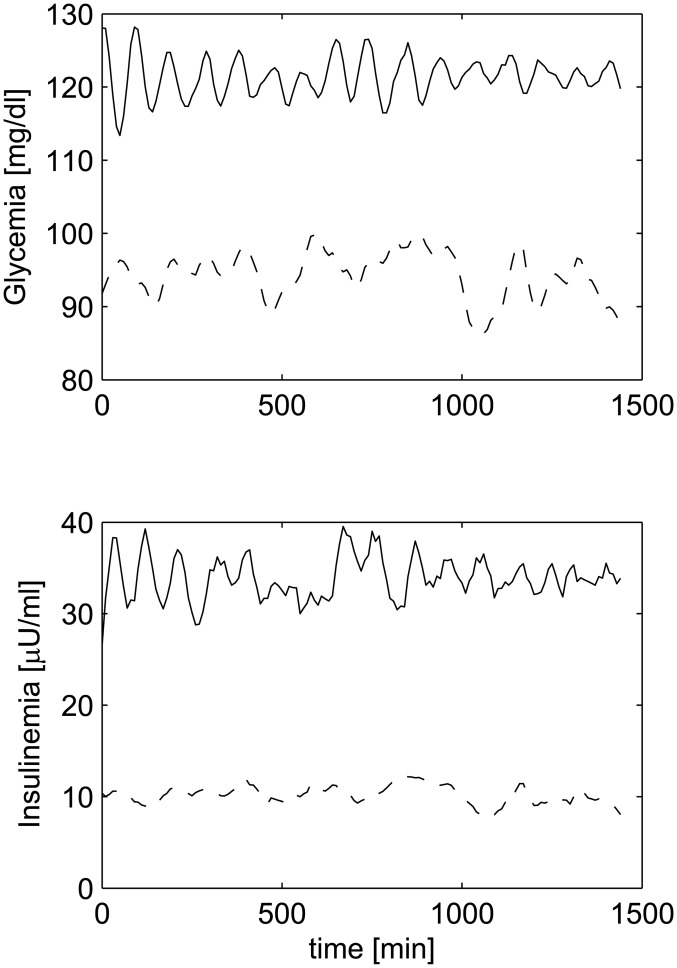
Glucose/insulin evolutions with and without enteral nutrition. Glucose (upper panel) and insulin (lower panel) evolution during a 24h period with (continuous line) and without (dashed line) enteral nutrition. Compare this figure with Fig 1 by Simon et al. [13].

Fig 8 shows the simulated glycemia (upper panel) and insulinemia (lower panel) of a virtual patient with (continuous line) and without (dashed line) enteral nutrition. It is evident here that spontaneous oscillations are dramatically amplified and synchronized by the constant input, exactly as shown in Fig 1 of Simon et al. [13].

### Ultradian oscillations in man: Sturis et al. 1991

The experiment performed by Sturis et al. [14] also investigates the phenomenon of low frequency (ultradian) oscillations in the insulinemia. In this case the oscillations are generated by three different patterns of exogenous I.V. glucose infusion, administered separately to the same subject over 24 h. The first infusion of 6 mg/kgBW/min was aimed at enhancing the spontaneous glucose/insulin oscillations (like in [13], but by means of intravenous glucose stimulation), allowing their natural frequency to be more easily and precisely determined. The constant infusion was then replaced by a sinusoidal glucose infusions with amplitude equal to 33% of the mean infusion rate and periods 20% greater (second infusion) or lower (third infusion) than the natural period detected during the first, constant infusion. The purpose of the experiment was to show the entrainment of insulinemia oscillations to the frequency of the administered glucose pattern.

In order to replicate the experiment, the glucose Eq (7) is modified as follows:

$$\begin{array}{ccc} \frac{dG(t)}{dt} & = & -k_{1}u\left(G\left(t\right)\right)+k_{2}I\left(t\right)G\left(t\right)+\frac{k_{3}\left(t\right)+k_{\text{ex}}\left(t\right)}{V_{G}},\\ k_{\text{ex}}\left(t\right) & = & {\bar{k}}_{\text{ex}}+\Delta_{\text{ex}}\sin\left(\frac{2\pi t}{T_{\text{ex}}}\right), \end{array}$$ (23)

where ${\bar{k}}_{\text{ex}}$ = 6 mg/kgBW/min. In this context:

- Δ_ex_ is set to zero in the first experiment, from which the endogenous period of oscillations *T*_end_ is estimated;
- $\Delta_{\text{ex}}=0.33{\bar{k}}_{\text{ex}}$ in the last two experiments, with *T*_ex_ = *T*_end_ + 0.2*T*_end_ and *T*_ex_ = *T*_end_ − 0.2*T*_end_ in the second and third experiments, respectively.

**Fig 9:**
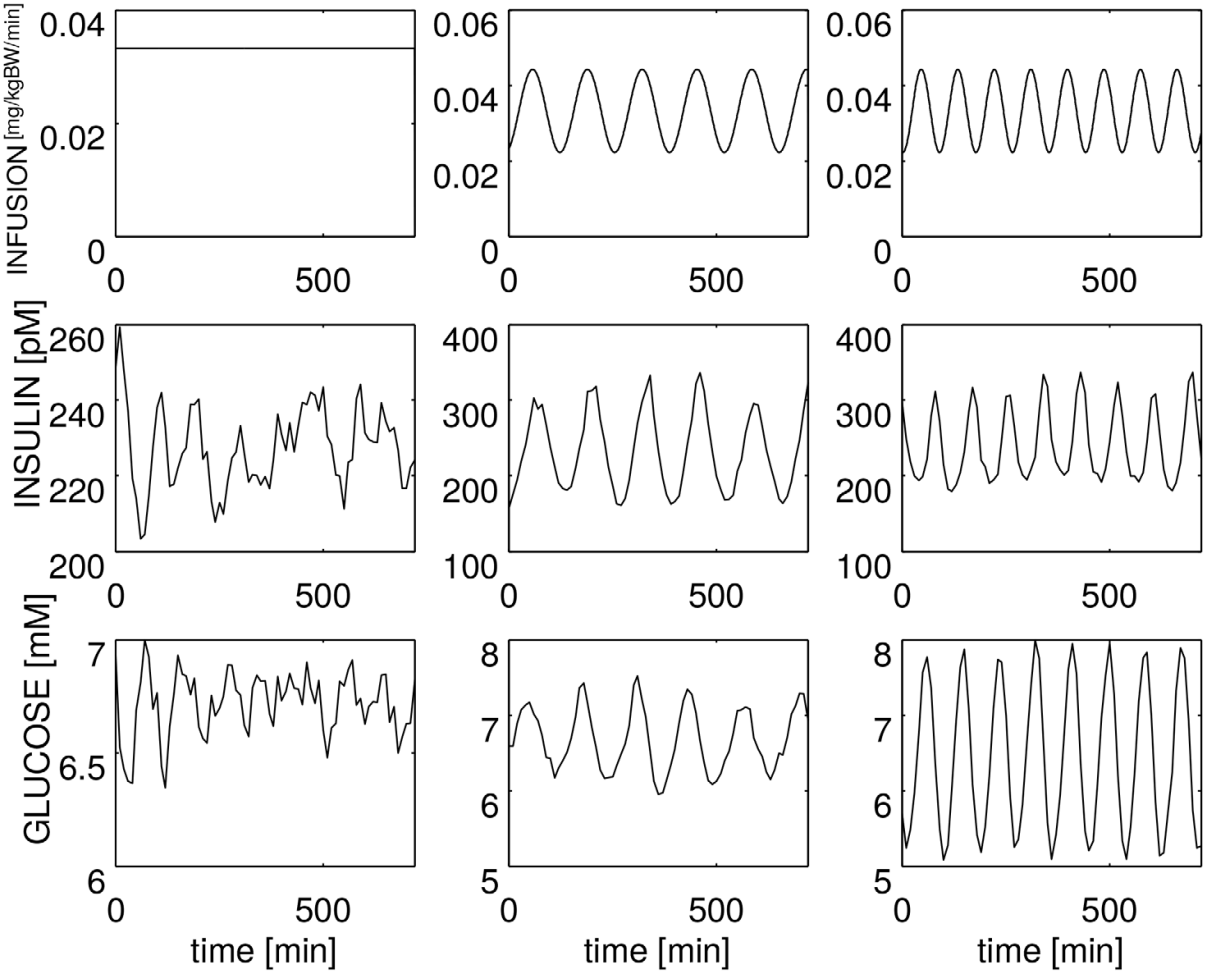
Glucose/insulin evolutions entrained by sinusoidal exogenous glucose administration. Glucose administration (top row), insulinemia (center row) and glycemia (bottom row) during a 720 min period. Compare this figure with Fig 1 by Sturis et al. [14].

Fig 9 shows the results of the simulation with our model: the three columns correspond to the three different experiments, the first row reports the exogenous IV glucose infusion, the second row reports insulinemia, the third row reports glycemia. It is clear that the model reproduces very well the entrainment phenomenon. Please note that the units for the exogenous glucose infusion are cc/hr according to Sturis et al. [14] and the equivalent value of 6 mg/kgBW/min for a generic 70 kg patient is 126 cc/hr, as shown in Fig 9, upper-left plot.

### High frequency oscillations in man: Pørksen et al. 2000

The experiment replicated here is the one performed by Pørksen et al. [15], aiming to investigate high-frequency insulinemia oscillations. Differently from previously described *in vivo* experiments, here the attention is focused on relatively large oscillations in insulinemia triggered by I.V. administration of very small amounts of glucose. Pørksen et al. showed in fact that an I.V. glucose administration of 6 mg/kgBW/min for 1 min every 10 min in healthy subjects induces large, clearly defined pulses of insulin secretion compared with the control state (i.e. no exogenous glucose administration), where irregular spontaneous high-frequency insulin oscillations occur. Moreover, when the train of glucose impulses has a period around 10 minutes, insulin peaks are entrained at the same frequency as the driving glucose boli.

In order to simulate this experiment, we modify the glucose equation adopted in the Sturis experiment Eq (23) as follows:

$$\begin{array}{cc} k_{\text{ex}}\left(t\right)=\left\{\begin{array}{cc} g_{\text{inf}}, & t\in [hT_{\text{ex}},hT_{\text{ex}}+T_{i})\\ 0, & t\in [hT_{\text{ex}}+T_{i},\left(h+1\right)T_{\text{ex}}), \end{array}\right. & h=0,1,\ldots , \end{array}$$ (24)

where *g*_inf_ (mmol/kgBW/min) is the exogenous glucose infusion rate, administered continuously during the first *T*_*i*_ minutes of the pulsing period *T*_ex_ (min).

According to the protocol used in [15], the pulsing period *T*_ex_ is fixed to 10 min, with *T*_*i*_ = 1 min, while two different values of *g*_inf_ have been considered (2 and 6 mg/kgBW/min), comparing the results with the control case, in which no exogenous glucose was administered. Glycemia and insulinemia are sampled every minute.

**Fig 10:**
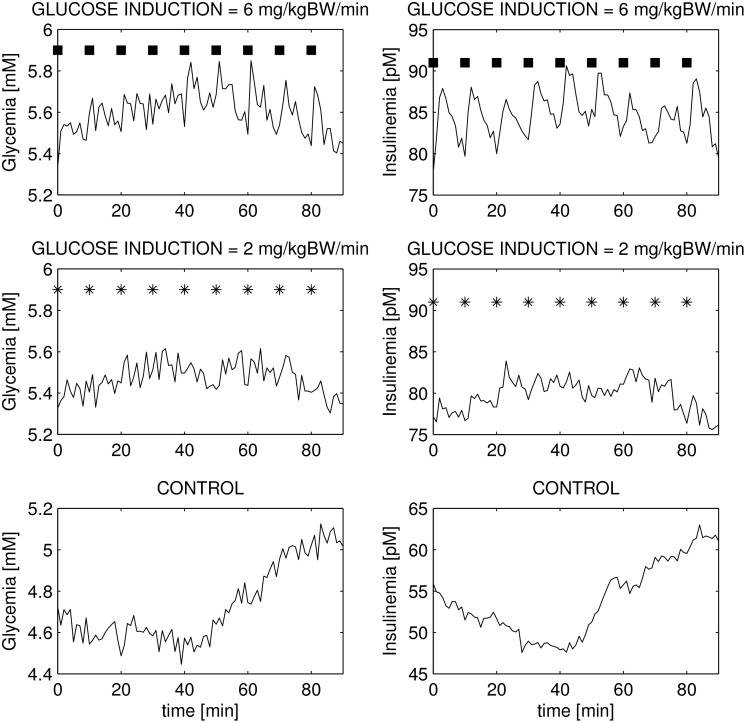
Glucose/insulin concentrations during high frequency minimal stimulation. Glucose (left panels) and insulin (right panels) evolution during a 1h30’ period with 6 (upper panels), 2 (center panels) and 0 (lower panels) mg/kg/min intravenous glucose administration. Compare this figure with Fig 1 by Pørksen et al. [15].

Results are plotted in Fig 10, where the three rows correspond to an administration of 6, 2 and 0 mg/kgBW/min, respectively, with the columns referring to glycemia and insulinemia respectively: these figures are to be compared with Fig 1 in [15].

In real experiments, the infusion of 6 mg/kgBW/min triggered marked pulsatile insulin oscillations at the same frequency as the input signal, while the corresponding glycemia neither showed the same entrainment, nor was visibly affected by the (small) impulses themselves. Moreover, inputs of 2 and 0 mg/kgBW/min do not induce appreciable responses in either glycemia or insulinemia. All of these features can be seen to be reproduced faithfully by the simulations from the model.

### Intra-venous glucose tolerance test (IVGTT) in man

**Fig 11:**
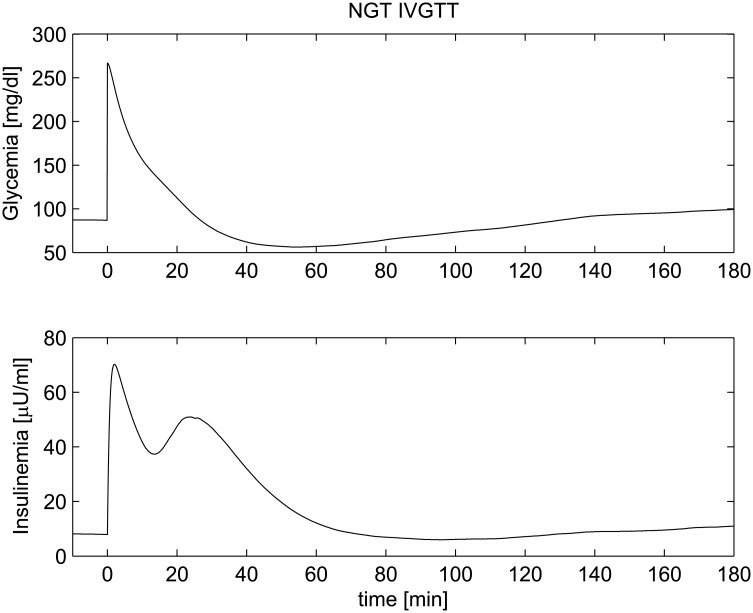
IVGTT experiment for an NGT patient. Simulated glycemia (upper panel) and insulinemia (lower panel) for a Normal Glucose Tolerance patient (NGT) during an Intravenous Glucose Tolerance Test (IVGTT).

**Fig 12:**
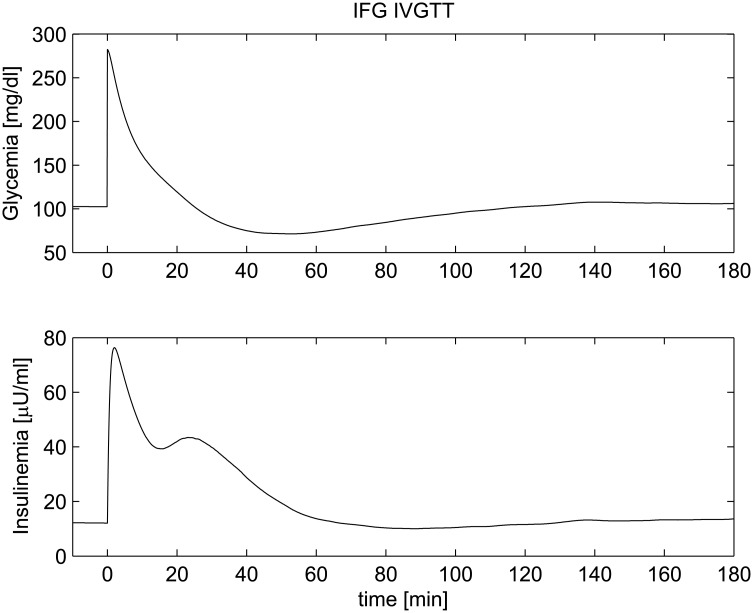
IVGTT experiment for an IFG patient. Simulated glycemia (upper panel) and insulinemia (lower panel) for an Impaired Fasting Glucose patient (IFG) during an IVGTT.

**Fig 13:**
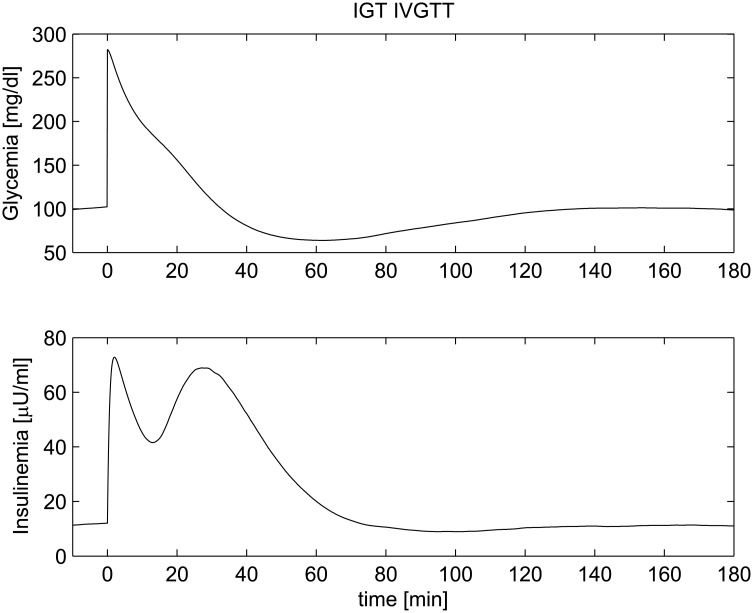
IVGTT experiment for an IGT patient. Simulated glycemia (upper panel) and insulinemia (lower panel) for an Impaired Glucose Tolerance patient (IGT) during an IVGTT.

**Fig 14:**
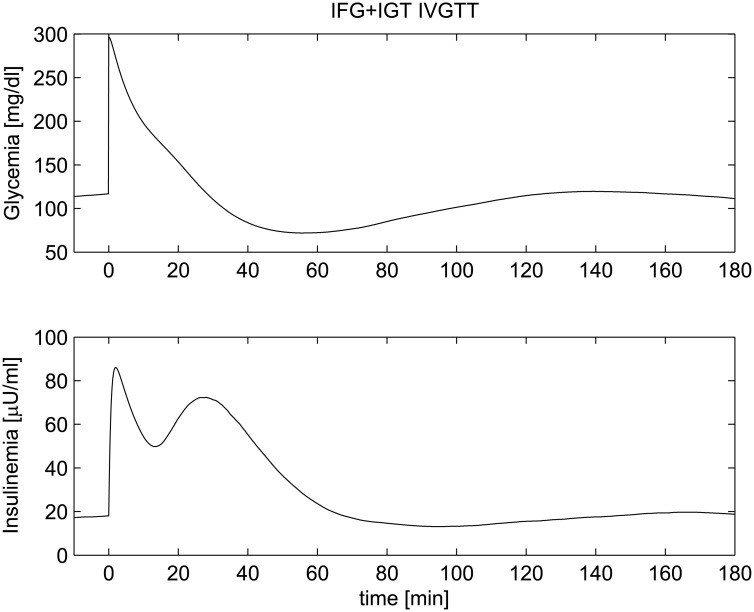
IVGTT experiment for an IFG+IGT patient. Simulated glycemia (upper panel) and insulinemia (lower panel) for a IFG+IGT patient during an IVGTT.

**Fig 15:**
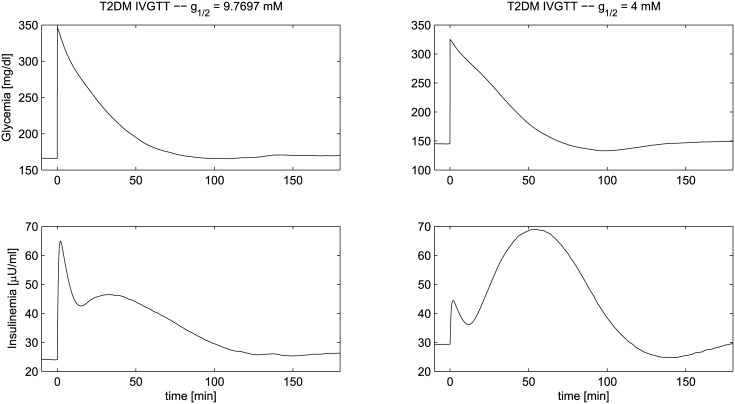
IVGTT experiment for an T2DM patient. Simulated glycemia (upper panels) and insulinemia (lower panels) for a Type-2 Diabetes Mellitus patient (T2DM) during an IVGTT. The left-side panels refer to a virtual patient whose thresholds distribution is the same as in the previous cases (*g*_1/2_ = 9.7 mM), while the right-side panels refer to a virtual patient with *g*_1/2_ = 4 mM.

The Intra Venous Glucose Tolerance Test (IVGTT) is a standard *in-vivo* experiment used to study insulin sensitivity and pancreatic response to a glucose stimulus. In a standard IVGTT, after a period (three days) of a standard composition diet (55% carbohydrate, 30% fat, 15% protein), with at least 250 g carbohydrates per day, subjects undergo the test in the morning after an overnight fast. A standard IVGTT (without either Tolbutamide or insulin injections) is accomplished by rapidly injecting (within one to four minutes) in an arm vein, at time 0 (0’), a solution containing glucose in a quantity ranging from 0.3 to 0.5g Glucose/kg Body Weight. Plasma glucose and insulin concentrations are measured from a contralateral arm vein at -30’, -15’, 0’, 2’, 4’, 6’, 8’, 10’, 12’, 15’, 20’, 25’, 30’, 35’, 40’, 50’, 60’, 80’, 100’, 120’, 140’, 160’ and 180’. In the present study the trends of plasma glucose and insulin concentrations over time, following an IVGTT with 0.36g glucose/kgBW, were simulated by the model (Eqs 1 to 12). In order to test the robustness of the model in reproducing this type of experiment, responses to the test of hypothetical subjects with different glucose tolerance states were simulated: a Normal Glucose Tolerance (NGT) subject, an Impaired Fasting Glucose (IFG) subject, an Impaired Glucose Tolerance (IGT) subject, an IFG+IGT subject and a Type 2 Diabetes Mellitus (T2DM) subject. The above conditions are determined on the basis of the glucose trend over time recorded following a 75g glucose load (oral glucose tolerance test, OGTT). The ability of the organism to maintain normal glucose homeostasis is indeed dependent on three tightly related processes: insulin secretion by pancreatic cells; stimulation of glucose uptake by splanchnic (liver and gut) and peripheral (primarily muscle) tissues; suppression of hepatic glucose output [70]. In the normal situation (NGT) fasting glycemia remains below 5.6 mM and glycemia returns to below 7.8 mM within 2 hours of the oral load. When a defect in glucose homeostasis arises, depending on the type of impairment, pathological conditions develop. The IFG condition is characterized by abnormal suppression of Hepatic Glucose Production (HGP) (central insulin resistance) and hence abnormal fasting plasma glucose (between 5.6mM and 6.9mM). IGT patients have insufficient tissue glucose uptake after load (peripheral insulin resistance) and present with 2-hour plasma glucose after oral glucose administration ranging between 7.8mM and 11mM. The associated IFG+IGT state occurs when both the above abnormalities are simultaneously present. The T2DM stage is characterized by substantial decompensation produced by insufficient insulin secretion in the face of either central or peripheral insulin resistance. T2DM is thus defined as either abnormal fasting plasma glucose (>7mM) or abnormal post-prandial glucose levels (>11mM), or both [71]. All the above conditions have been reproduced by suitably changing the parameter values used for the NGT scenario (the same set of model parameters adopted for previous *in vivo* experiments reported in Table 1), as reported in Table 4. Figs 11 to 15 show plasma glucose and insulin concentrations over time following the glucose bolus in the five simulated normal and pathophysiological conditions. In Fig 11 the time-course of the two state variables is reported for NGT: this time-course is very close to the average glycemia and insulinemia time course observed during IVGTT in healthy patients [59]. Following a sudden increase in glycemia, insulin is secreted in two phases: a first phase (in the very first few minutes after the beginning of the experiment) and a second phase (between 10 and 20 minutes after the glucose bolus). Insulin is secreted consistently with observed glucose levels and normal glycemia is soon restored at basal glucose levels. The model parameter *k*_2_ represents the (peripheral) insulin sensitivity index, which in normal subjects is of the order of 1 × 10^−4^ [57, 59]. Parameter *k*_3_ represents instead HGP as a consequence of glycogenolysis and gluconeogenesis. In absence of central, hepatic insulin resistance, high plasma insulin levels suppress both mechanisms; this means that an IFG patient should present with increased HGP, i.e. with a higher *k*_3_ value, set therefore in the simulations to 1.6 times the NGT reference value. Fig 12 reports the trend of plasma glucose and insulin concentrations following an IVGTT in an IFG patient. Apart from a fasting glucose concentration higher than that observed in normal subjects (caused by a higher value of the parameter *k*_3_), the two profiles are not dissimilar from those characterizing NGT subjects. Fig 13 shows an IGT patient, whose insulin sensitivity index *k*_2_ was set at 65% of the normal value. In this case glucose still eventually decreases towards normal values as a consequence of the higher amounts of secreted insulin (see the bottom panel of Fig 13).

**Table 4:** Parameters varying values for diseased patient undergone an IVGTT.

Par.	**NGT**	**IFG**	**IGT**	**IFG+IGT**	**T2DM**
*k*_2_	1.4 × 10^−4^	1.4 × 10^−4^	1.4 × 10^−4^ ⋅ 0.65	1.4 × 10^−4^ ⋅ 0.65	1.4 × 10^−4^ ⋅ 0.3
${\bar{k}}_{3}$	0.01	0.01 ⋅ 1.6	0.01	0.01 ⋅ 1.6	0.01 ⋅ 1.6
*μ*(*ρ*_*n*_)	6.5 × 10^−3^	6.5 × 10^−3^ ⋅ 0.5	6.5 × 10^−3^ ⋅ 0.5	6.5 × 10^−3^ ⋅ 0.25	6.5 × 10^−3^ ⋅ 0.05
$\mu ({\bar{D}}_{n})$	3 × 10^−3^	3 × 10^−3^	3 × 10^−3^	3 × 10^−3^	3 × 10^−3^ ⋅ 0.3
*g*_1/2_	9.7697	9.7697	9.7697	9.7697	9.7697 and 4

The problem of the effects of the two different types of insulin resistance on insulin secretion has been tackled in different studies. Most of the studies aiming at estimating insulin secretion capacity performed OGTTs, while a limited number of studies performed both OGTTs and IVGTTs. Findings are controversial. Some studies report a decrease in Acute Insulin Response (AIR) after IVGTT (that is a decreased first-phase insulin secretory response) in both isolated IFG and IGT compared with NGT [72–74]. Investigations in Pima Indians have shown that, with regard to the AIR after intravenous glucose injection, IFG subjects have more severe defects than those with isolated IGT [75]. However, Faerch et al. [76] found that absolute first-phase insulin secretion during IVGTT was decreased in IFG but not in IGT compared with NGT. In the work by Abdul-Ghani, Tripathy and De Fronzo [77], subjects with isolated IFG manifested a decrease in first-phase insulin secretory response to intravenous glucose and subjects with IGT had severe defects in both early- and late-phase insulin responses to intravenous glucose. However it is known that in NGT subjects, the amount of insulin secreted in response to glucose is inversely correlated with peripheral insulin sensitivity [78–81]. Reduced insulin sensitivity, therefore, determines an increased plasma insulin response to any given glucose stimulus. Simulations in Figs 12 and 13 show this kind of mechanism: in the early stages of these pathophysiological conditions the pancreas reacts to higher, sustained plasma glucose levels by compensatory increased insulin secretion: normal glucose tolerance, when still maintained, becomes critically dependent on the *β*-cell’s ability to increase its secretion in an attempt to offset the defects in insulin action [82]. Thus, in patients with impaired glucose tolerance and in diabetic patients with mild fasting hyperglycemia (6.1–7.8 mM), plasma insulin response to glucose is uniformly increased [82]. For both IFG and IGT, the parameter *μ*(*ρ*_*n*_) was decreased (down to 50% normal) in order express the initial decrement of *β*-cell efficiency (the parameter *μ*(*ρ*_*n*_) represents the average maximal loading potentiation rate). The increased insulin response is however still produced by the system, through the recruitment of a larger number of secretory units. Fig 14 shows a patient with both types of insulin resistance (parameters *k*_2_, *k*_3_ and *μ*(*ρ*_*n*_) were all modified as before). Here the second phase of insulin secretion is lower than that observed for isolated IGT, highlighting a progressive deleterious effect of chronic hyperglycemia on insulin secretion and insulin action [78], a concept that has been referred to as glucose toxicity. Finally Fig 15 shows two different scenarios for a T2DM patient, corresponding to two different hypothetical mechanisms of disease. In both scenarios, besides more severe defects of the same type as in the IFG+IGT condition, the parameter $\mu (\bar{D_{n}})$ is decreased by 70%. This parameter represents the basal insulin packet size. A decrement in *μ*($\bar{D}$) impacts on insulin secretion: under this hypothesis the long hyperglycemia has compromised *β* cell functionality (the pancreas produces less insulin), higher than normal glycemia levels are observed during the IVGTT and a longer time is necessary to return to basal conditions. That these defects occur is undisputed in the literature. The second scenario however introduces an additional variation, a decrement of the parameter *g*_1/2_ shifting the glycemic threshold distribution of the controllers to the left. A decrease in this parameter means a faster, more complete recruitment of *β*-cells at lower glycemias: under this hypothesis on the mechanism of disease, the first phase response is essentially missing and a more sustained second phase is necessary to face the sustained higher glycemias.

## Discussion

As presented at length in the Introduction, pancreatic insulin secretion has been the object of many explorative experiments and of several modelling attempts. The model proposed in the present work improves a previously published similar model [16], with changes to the equations and with a thorough revision of the parameter values based upon the original Grodsky results. The main goal of this model is to provide a unified explanation of an array of diverse experimental procedures.

The basic paradigm of this model is that in the pancreas a multitude of similar, but not identical, controllers react to the sensed plasma glucose, which acts as the single “coupling” signal. In this way, our model does not need to hypothesize either a glucose-independent pancreatic pacemaker, nor a dependency of insulin secretion on the rate of change of glucose concentration [6, 30–32, 46, 61]. The controllers are assumed to share the same basic response mechanism, reminiscent of the way other excitable cells and tissues in the body work (e.g. neurons or cardiac muscles cells [24, 25]): each controller fires when circulating glucose concentration reaches the critical threshold for that controller; upon firing, the controller releases a discrete packet of insulin into the circulation; after having fired, the controller enters a relative refractory period, with refractoriness progressively attenuating until the pristine, excitable state is reached and the controller may fire again. The metabolic loop is closed, in the model, by hypothesizing simple mechanisms of insulin removal from the circulation and of glucodynamic insulin effect.

While hundreds of thousands of controllers are represented in the model, the behaviour of the whole system is determined by specifying only few controller population metaparameters: these are the characteristics of the distributions from which each controller’s parameters are randomly sampled (glycemia firing threshold; rapidity of return to normal excitability determining the length of the refractory period; rate of increase of insulin packet size upon persisting hyperglycemia, etc.). The entire model behaviour depends, in this way, on a small number of parameters and metaparameters (see Tables 1 and 2), notwithstanding its structural complexity.

The major contribution of the present work is to show that a model of this type, using a single plausible parameter set, may faithfully reproduce a wide array of heterogeneus, morphologically rich experimental results obtained in man, and that, by introducing modest changes in parameters, other diverse and characteristic experimental results from ex-vivo animal preparations are also faithfully reproduced.

It should be appreciated that, while it is rather easy to justify qualitatively certain effects on the basis of a plausible mechanistic interpretation (e.g. it is rather obvious that introducing some delay into the glucodynamic effect of insulinemia could determine slow oscillations in glycemia and insulinemia itself), building a single model, which can quantitatively reproduce, with a single set of parameter values, a whole collection of different and richly structured experimental data sets is a more ambitious endeavour. In fact, no such model has been proposed so far, to the best of our knowledge.

The model exhibits, first of all, both high-frequency and slow-frequency insulinemia oscillations. High-frequency oscillations appear to be caused by firing-refractoriness-recuperation cycles over the subpopulation of controllers whose thresholds are below current glycemias. Low-frequency oscillations, conversely, appear due to the negative-feedback delay of secreted insulin inducing increments of tissue glucose uptake.

The model is consistent with clinical observations in predicting an accentuation and synchronization of low-frequency insulinemia oscillations, when glycemia is constantly raised (by meals or by endo-venous constant glucose infusion). This amplification of response is likely due to the increased recruitment of secretory units and should in fact disappear or be significantly reduced in those diseases (like diabetes mellitus) where the possibility of recruiting a large number of functional controllers is reduced or absent: this also is in accord with experimental observations.

In this context it should be underscored that, while many models could associate insulin oscillations with glucose oscillations, the present model predicts accentuated insulin oscillatory behaviour corresponding with constantly raised glycemias.

It has been shown that the impairment of the ability of exogenous glucose stimuli to entrain insulin oscillations is a highly sensitive manifestation of *β*-cell secretory dysfunction. This has been clearly assessed for low-frequency oscillations [83–85] in diabetic and prediabetic patients, as well as for high-frequency oscillations [86, 87] where comparisons have been carried out on healthy subjects versus T2DM patients, with both large [87] and small [86] glucose pulses. Further experiments, made by replacing glucose with arginine (which successfully entrained insulin oscillations in T2DM patients as well) showed that the loss of entrainment is likely to be a glucose-specific *β*-cell defect [88]. Loss of glucose entrainment has, in fact, also been found in *in vitro* experiments on isolated perfused pancreata of Zucker diabetic fatty rats [42]. The model agrees with the experiments by Pørksen and colleagues [15]: when minimal amounts of glucose (undetectable by observation of glycemia) are administered periodically, high-frequency insulin pulsatility is clearly accentuated and is made to synchronize with the frequency of glucose administration, even when this is somewhat higher or lower than its natural frequency. The experiments by Sturis et al. [14], involving entrainment to an oscillating glycemic signal, are also well replicated.

The curves obtained by the model, upon simulated I.V. glucose administration, are typical of what is clinically observed in IVGTT experiments. In particular, both a primary and a secondary insulin response are evident and the variation of the shape of the insulinemia response closely mimics what is observed in the clinical setting, as the parameters quantifying tissue insulin sensitivity, liver glucose output, and pancreatic secretory capacity are varied to reflect Normal Glucose Tolerance, Impaired Fasting Glycemia, Impaired Glucose Tolerance, IFG+IGT and finally Type 2 Diabetes Mellitus. It is of interest, in this context, that the model predicts differently shaped curves depending on whether a shift to the left in the controllers threshold distribution occurs or does not occur as prediabetes worsens and T2DM becomes apparent. This indicates a potential theory-driven direction for experimentation: since the curves commonly observed in T2DM patients are more similar to the prediction made by the present model incorporating a left shift of controller thresholds, does this left-shift actually occur in patients pathophysiology?

The model is able to near-perfectly reproduce the increasing insulin release spikes observed upon progressively increasing (staircase) glycemic stimulation of *ex-vivo* rodent pancreas preparations. Moreover, the model reproduces very well the initial spike, fall and progressive increase connected with the sudden start of constant glycemic stimulation, and also quantitatively agrees with the observations as progressively higher levels of glycemia are tested.

The shape and size of the successive peaks determined by discontinuous glucose administrations are also perfectly reproduced: here it is worth noticing that the peak corresponding to the second (re-started) infusion is substantially larger than the peak at the start of the first infusion, as is indeed the case experimentally. The responses to ramp-and-cease as well as to progressively increasing glycemia stimulations are also in close accord with the experimental results. The varied morphology of these response curves derives in a straightforward fashion from the interplay of controller recovery, controller recruitment and potentiation.

The fact that a single model, with a single (species-specific) set of parameters, is able to replicate such a diverse array of experimental procedures, down to the minute characteristics of spikes and troughs, of shapes of increasing ramps, of coherent frequency and amplitude responses to non-oscillating or minimal inputs, justifies the conclusion that the model captures the essential mechanics of the pancreatic control of glycemia by means of insulin secretion. This close agreement with a variety of experiments is the result of the model structure, which closely replicates the actual anatomical and physiological behaviour of the organ. A limitation of the present model is therefore that its structure is rather complex, even though the (meta-)parameter set is small. In the future it may be possible that other, more concise formulations will attain the same level of fidelity.

For the moment, however, the proposed model appears able to account for such a diversity of physiological and clinical manifestations that strong support is provided to the theory that neither pancreatic pacemakers, nor single controller cycling (e.g due to oscillating *β*-cell glycolysis), nor differential controlling (i.e. ability of the controllers to sense rates of change of glycemia) need to be invoked in order to explain pancreatic insulin secretion.
